# Successful delivery of docetaxel to rat brain using experimentally developed nanoliposome: a treatment strategy for brain tumor

**DOI:** 10.1080/10717544.2016.1253798

**Published:** 2017-02-06

**Authors:** Tapan Kumar Shaw, Dipika Mandal, Goutam Dey, Murari Mohan Pal, Paramita Paul, Samrat Chakraborty, Kazi Asraf Ali, Biswajit Mukherjee, Amal Kumar Bandyopadhyay, Mahitosh Mandal

**Affiliations:** 1 Department of Pharmaceutical Technology, Jadavpur University, Kolkata, West Bengal, India,; 2 School of Medical Science and Technology, Indian Institute of Technology Kharagpur, Kharagpur, West Bengal, India, and; 3 Dr. B. C. Roy College of Pharmacy and Allied Health Sciences, Dr. Meghnad Saha Sarani, Bidhan Nagar, Durgapur, West Bengal, India

**Keywords:** Blood–brain barrier, nanoliposomes of Docetaxel, glioma, C6 cells, brain distribution

## Abstract

Docetaxel (DTX) is found to be very effective against glioma cell *in vitro*. However, *in vivo* passage of DTX through BBB is extremely difficult due to the physicochemical and pharmacological characteristics of the drug. No existing formulation is successful in this aspect. Hence, in this study, effort was made to send DTX through blood–brain barrier (BBB) to brain to treat diseases such as solid tumor of brain (glioma) by developing DTX-loaded nanoliposomes. Primarily drug-excipients interaction was evaluated by FTIR spectroscopy. The DTX-loaded nanoliposomes (L-DTX) were prepared by lipid layer hydration technique and characterized physicochemically. *In vitro* cellular uptake in C6 glioma cells was investigated. FTIR data show that the selected drug and excipients were chemically compatible. The unilamellar vesicle size was less than 50 nm with smooth surface. Drug released slowly from L-DTX *in vitro* in a sustained manner. The pharmacokinetic data shows more extended action of DTX from L-DTX in experimental rats than the free-drug and Taxotere®. DTX from L-DTX enhanced 100% drug concentration in brain as compared with Taxotere® in 4 h. Thus, nanoliposomes as vehicle may be an encouraging strategy to treat glioma with DTX.

## Introduction

Astrocytoma (commonly known as glioma) is most prevalent among three different types of brain tumors, namely astrocytomas, oligidendrogliomas and oligoastrocytomas, in adults. This aggressive malignant form of cancer accounts for ∼45–50% of all primary tumors resulting in death of patients within a couple of years (Guo et al., 2011; Nance et al., 2014). The characteristic features such as lack of sharp border, infiltration ability of the tumor cells in the brain of glioma as well as their wide distribution restrict their treatments by surgery and radiotherapy (Guo et al., 2011). Further, due to the strategic location of the blood–brain barrier (BBB) that allows a selective transport of drugs into the brain, chemotherapy becomes an auxiliary treatment for malignant glioma. In the last few decades, many drugs have been or being explored for the treatment of glioma. Most of them including docetaxel (DTX) are large hydrophobic molecules, which are unable to cross the BBB easily (Asperen et al., 1997) and may become an effective candidate for efflux by various efflux pumps governed by BBB as well as tumor cells (Beaulieu et al., 1997).

Docetaxel (DTX) is a complex diterpene alkaloid, isolated from the bark of *Texas baccata,* congener of paclitaxel. It has an efficient antineoplastic effect against a wide spectrum of solid tumors, such as ovarian, breast and lung cancer. It is found to be effective in the treatment of glioma *in vitro* but its *in vivo* efficacy is highly compromised due to its poor aqueous solubility and high molecular weight (Banks, 2009; Liu et al., 2011; Tan et al., 2012). Therefore, suitable design and development of appropriate vehicle for the transport of therapeutic payload is of prime importance in order to develop an effective therapy against glioma. In this context colloidal drug carrier especially nanoliposomes have gained significant interest among the researchers around the globe. (Jain, 2012; Zhang & Zhang, 2013; Hao et al, 2015; Sonali et al., 2016a; Sonali et al., 2016b; Sonali et al., 2016c).

Liposomes, the small spherical vesicle with single or multiple lipid bilayers, made from natural and/or synthetic lipids have been widely exploited due to their unique characteristics such as high biocompatibility, biodegradability, and non-immunogenicity (Laouini et al., 2012; Akbarzadeh et al., 2013). They usually improve biodistribution and pharmacokinetic profile of the therapeutic payload by sustained drug release from the formulation and minimizing the toxicity of the chemotherapeutics by their selective accumulation in the target area (Chang & Yeh, 2012; Akbarzadeh et al., 2013). Moreover, liposomes are able to accommodate both hydrophilic (in the aqueous core) drug and hydrophobic (in the lipidic bilayers) drug. Thus a liposomal drug delivery system can be used to increase the solubility of hydrophobic drugs and can protect them from metabolism in the body fluid (Allen & Moase, 1996; Irache et al., 2011; Akbarzadeh et al., 2013). However, optimized size of liposomal formulation is a critical aspect in order to develop efficient therapeutics against glioma. It has been reported in the literature that a nanosize (<200 nm) drug carrier can exploit the advantages of leaky vasculature associated with primary brain cancer for transport of therapeutic payload within the tumor easily through a common mechanism known as enhanced permeability and retention effect (EPR) (Kobayashi et al., 2014).

The marketed formulation of DTX (Taxotere®) contains a solubilizing vehicle which consists of 50:50 mixtures of tween 80 and dehydrated ethanol; and tween 80 is responsible for certain adverse effects such as severe hypersensitivity reactions, anaphylaxis, hyperlipidemia, abnormal lipoprotein patterns, and aggregation of erythrocytes (Tije et al., 2003). Further, infusion of DTX for a prolong period of time may sometimes cause cumulative fluid retention, and peripheral neuropathy (Sanchez-Moreno et al., 2012).

Administration of DTX requires a long infusion period (typically 3–24 h) and patients are generally required to be admitted in hospitals overnight resulting additional inconvenience. Therefore, incorporation of DTX into liposomal formulation results in a sustained release formulation, which could be administered easily through intravenous route and also use of tween 80 can be avoided, resulting improve patient compliance and better therapeutic outcome. Innovations of the present study focused on the development of nanosize (<100 nm) homogeneously distributed liposomes containing DTX using common materials with a simple technology. The developed formulation was able to cross blood brain barrier successfully to provide adequate concentration of drug in brain for a longer period by releasing the drug in a sustained manner. Comparable to the commercial formulation containing tween 80, further our formulation was devoid of tween 80 related toxicity.

Few similar recent important investigations (Hao et al., 2015, Sonali et al., 2016b, Sonali et al., 2016c) also developed nanoformulations with receptor targeting agents. However, they were constituted with absolutely different constituents such as d-α-tocopheryl polyethylene glycol 1000 succinate (TPGS), poly-lactic-co-glycolic acid (PLGA). Further, Sonali et al. (2016c) developed micelles. Hao et al. (2015) developed multifunctional nanoparticles and Sonali et al. (2016b) developed theranostic liposomes.

Advantages of our studies over those studies are that we have developed simple nanosize liposome with very common constituents such as phospholipid and cholesterol without any receptor targeting agent, using a very simple technology. Our formulations were much smaller than the size of the liposomes and nanoparticles reported in those studies. However, our formulations were little larger than the micelles, but well below 100 nm in size. Further, it provided much slower drug release with a more sustaining effect as compared to the above three formulations. Moreover, IC_50_ value of our formulation was considerably lower and the formulations were internalized well by the C6 glioma cells. The major disadvantage of the targeted delivery is the saturation of the receptor by the targeting agent. Our formulation is also devoid of such problem, and thus would deliver the drug more effectively for a prolonged period of time across the BBB to the brain.

Therefore, the objective of the present study was to develop nanoliposomal formulation of DTX and to establish its successful delivery in the brain. This may be beneficial for the treatment of brain cancer such a glioma.

## Materials and methods

DTX was obtained as gift sample from Fresenius-Kabi Oncology Ltd. (Kalyani, West Bengal, India). Cholesterol (CHL) and chloroform were purchased from Merck (Mumbai, India) and soya-l-α-lecithin (SPC), fluorescein isothiocyanate (Isomer I) (FITC), Dulbecco’s Modified Eagle’s Medium (DMEM) containing 10% fetal bovine serum (FBS) and antibiotics (10,000 U/L penicillin and 10 mg/L streptomycin) were purchased from HiMedia Laboratories Pvt. Ltd (Mumbai, India). NaHCO_3_ was purchased from Invitrogen Corporation (Carlsbad, CA), butylated hydroxy toluene (BHT) was purchased from Qualigens Fine Chemicals (Mumbai, India). 4′,6-Diamidino-2-phenylindole) (DAPI) and tetrazolium dye 3-(4,5-dimers dimethylthiazol-2-yl)-2,5-diphenyltetrazolium bromide (MTT) were purchased from Sigma-Aldrich (St. Louis, MO). C6 glioma cells of rats were purchased from National Center for Cell Science (Pune, India). Marketed formulation of DTX (Taxotere® injection 20 mg vial) was purchased from M/s Adeline, Kolkata, India (batch no. 4F170A, invoice no. 31375). All other chemicals and reagents used were of analytical grade.

### Experimental animals

Male Sprague-Dawley rats of body weight 160 ± 20 g were used for this study. Animals were obtained from animal house, Department of Pharmaceutical Technology, Jadavpur University, Kolkata, India. The animals were acclimatized to the laboratory condition at 22 ± 1 °C and humidity of 60 ± 10% for 7 days prior to the actual experiment and they had free access to water and food (Dey et al., 2016). The animals were maintained at 12 h dark/light circle. The animal experiments were conducted with the prior approval of the Institutional Animal Ethical Committee (IAEC), Jadavpur University, Kolkata, by strictly following the guidelines of the IAEC. The animals were deprived of food for 12 h with a free access of water before administration of different formulations.

### Drug-excipients interaction study

In the present study, we have investigated the drug-excipients interaction by Fourier transform infrared (FTIR) spectroscopy (Dey et al., 2016). The pure components of the experimental formulations (i.e. DTX, CHL and SPC, a mixture of CHL, SPC and a mixture of drug with CHL, SPC), the optimized formulation containing DTX (L-DTX) and formulation without DTX (B-DTX) (both the formulations were in the lyophilized forms) were mixed separately, with infrared grade potassium bromide (KBr) in a ratio of 1:100. Pellets were prepared using KBr press at a pressure of 5.5 metric ton. The pellets were then scanned using FTIR spectrophotometer (Jasco, FTIR 4200, Japan).

### Preparation of nanoliposomes (NLs)

DTX loaded NLs (L-DTX) were prepared by lipid layer hydration method as reported earlier (Dey et al., 2016). Requisite weights of different components of NLs such as SPC, CHL, DTX (Table 1) were taken into a 250 mL round bottom flask and were dissolved in 10 mL chloroform. The resultant mixture was mixed by gently-shaking the content in a rotary vacuum evaporator (Rotavap, model: PBU-6, Superfit Continental Pvt. Ltd., Mumbai, India) fitted with an A3S aspirator (Eyela, Tokyo Rikakikai Co. Ltd., Taguig City, Philippines) and circulating water bath (at 4 °C) (Spac-N Service, Kolkata, India) and the chloroform was evaporated at 40 °C in the water bath. It was then kept overnight in a vacuum desiccator. The thin film thus obtained was hydrated in PBS (pH,7.4) for 60 min at 60 °C at 160 rpm and sonicated at about 30 ± 3 KHz using a sonicator (Trans-o-Sonic, Mumbai, India) for 60 min. It was then preserved overnight at 4 °C. The suspension was centrifuged in a cold centrifuge (3K30 Sigma Lab Centrifuge, Merrington Hall Farm, Shrewsbury, UK) at 16 000, rpm at 4 °C for 60 min to obtain the liposomes and collected in a petridish; and freeze-dried using a laboratory freeze-drier (Laboratory-Freeze Dryer, Instrumentation India Ltd., Kolkata, India) for getting a dried mass of the sample.

**Table 1.** Formulation composition, % yield, drug loading and loading efficiency of different formulations.

Formulation code	DTX:SPC:CHL (by weight)	Yields %*	% of drug loading*	Loading efficiency %*
NL1	1:5:5	42.03 ± 4.19	2.6 ± 0.45	28.61 ± 5.05
NL2	1:10:5	69.21 ± 4.75	5.48 ± 0.5	87.81 ± 7.82
NL3	1:15:5	49.89 ± 2.86	1.86 ± 0.5	70.26 ± 11.04

*Data show mean ± standard deviation of three different formulations or three different experiments in triplicate (where applicable).

NLs without DTX (B-DTX) were prepared by the same method without the addition of DTX during preparation. Fluorescent NLs containing DTX (F-DTX) for cellular uptake study were prepared by the same method of L-DTX preparation except that addition of 100 μL of 0.4% (w/v) FITC, as a fluorescent marker, dissolved in a mixture of chloroform and ethanol (3:1 v/v) (Sinha et al*.,* 2013) during the initial mixing of the components in chloroform.

### Vesicle characterization

#### DLS study

The average vesicle size, polydispersity index (PDI) and zeta potential of L-DTX were determined by dynamic light scattering (DLS) technology in a Zetasizer Nano ZS90 (Malvern Instruments, Malvern, UK) at 25 °C using a standard method of analysis (Dey et al., 2016).

#### Surface morphology study by field emission scanning electron microscopy (FESEM)

FESEM of optimized NLs was done by using electron microscope (Model-JSM-6700F; JEOL, Tokyo, Japan). Lyophilized formulation was spread on to a carbon tape over a stub and a platinum coating of about 5 nm was applied at an accelerating voltage of 10 kV and 10 mA current with the help of a platinum coater (JEOL, Tokyo, Japan).

#### Cryogenic-transmission electron microscopy (Cryo-TEM) study

Further, morphology, rigidity and lamellarity were confirmed by Cryo-TEM as reported earlier and images were captured on a BM-Eagle 4 k × 4 k CCD camera (FEI Company, Eindhoven, The Netharlands) and the final pixel size was 1.89 Å (Helvig et al., 2015; Dey et al., 2016).

#### Yield percentage

Yield of each formulation batch was determined to know the amount of NLs obtained with respect to the total amount of raw materials used for the formulation. The completely dried formulation was weighed after a batch run and percentage yield was then determined by the following equation:

$$\mathrm{Percentage}\;\mathrm{yield}\;=\frac{\mathrm{Amount}\;\mathrm{of}\;\mathrm{NLs}\;\mathrm{obtained}}{\mathrm{Total}\;\mathrm{amount}\;\mathrm{of}\;\mathrm{components}\;\mathrm{in}\;\mathrm{the}\;\mathrm{formulation}}\times 100$$

#### Percentage of drug loading and loading efficiency percentage

Requisite quantity of NLs (2 mg) was lysed by sonication followed by vortex in acetonitrile (HPLC grade) and DTX was separated out by centrifugation at 10 000, rpm for 10 min. The absorbance of supernatant was measured at 230 nm in an ultraviolet-visible spectrophotometer (Advanced Microprocessor UV-Visible single beam, Intech 295, Andhra Pradesh, India). The same procedure was repeated for formulation without drug (B-DTX) to get absorbance to nullify the effect of excipients on DTX absorbance readings by subtracting the readings of the absorbance of B-DTX from those of L-DTX. The percentage drug loading and loading efficiency were calculated from following equations as mentioned earlier (Dey et al., 2016).

$$\mathrm{Percentage}\;\mathrm{of}\;\mathrm{drug}\;\mathrm{loading}=\frac{\mathrm{Amount}\;\mathrm{of}\;\mathrm{DTX}\;\mathrm{in}\;\mathrm{NLs}}{\mathrm{Amount}\;\mathrm{of}\;\mathrm{NLs}\;\mathrm{taken}}\times 100$$

$$\text{Percentage loading efficiency }=\frac{\text{Practical loading \%}}{\begin{array}{c} Theoretical\;loading\;\% \end{array}}\times 100$$

#### In vitro drug release and drug release kinetic study

Comparative *in vitro* drug release behavior of the optimized formulation (L-DTX) with the marketed formulation (Taxotere®) and free-drug suspension, consisted of 2 mg/mL of DTX in a mixture of 520 mg of tween 80 and 13% ethanol (Dou et al., 2014) was investigated by dialysis method (Dey et al., 2016). Briefly, phosphate-buffer saline pH 7.4 (PBS), 50 ml containing 0.5% (w/v) sodium lauryl sulfate (SLS) (Hu et al., 2012) was taken as drug release media in a borosilicate glass beaker of 100 ml capacity. Accurately weighed (5 mg) formulation (lyophilized) and an equivalent amount of free-drug suspension; and Taxotere®; were reconstituted with 1 mL drug release media and poured into a dialysis bag (molecular weight cut-off 12–14 kDa). The bag containing formulations was made and immersed centrally into the release media in a glass beaker (100 ml) with the help of a glass rod. The beaker was placed on a magnetic stirrer and maintained the content of the beaker for a rotation of 300 rpm using a magnetic bead. At different predetermined time intervals for 48 h, drug release medium (1 mL) was withdrawn and replaced with 1 mL fresh drug release media. The absorbance of the samples was measured in a spectrophotometer at *λ*_max_ of 229 nm. The different concentrations were calculated from the calibration curve of DTX in PBS containing 0.5% SLS.

To understand *in vitro* DTX release kinetics from L-DTX formulation, the drug release data was analyzed by different release kinetic models such as zero-order, first-order, Higuchi kinetics, Korsmeyer–Peppas model and Hixon–Crowell model for the highest correlation coefficient value (R^2^) (Pattnaik et al., 2012).

#### In vitro cell viability assay

Cell viability assay in rat C6 glioma cells was performed to monitor growth inhibitory potential or cytotoxicity of different formulations of DTX such as, L-DTX, B-DTX, Taxotere® and free DTX solution, by MTT assay (Barth, 1998; Dey et al*.,* 2015). These cells were grown in DMEM containing 10% FBS, NaHCO_3_ and antibiotics (10 000 U/L penicillin and 10 mg/L streptomycin). C6 cells were maintained in T-25 culture flask at 37 °C in CO_2_-incubator (Heraeus Hera Cell, Burladingen, Germany). Briefly the method is as follows:

C6 glioma cells (2.5 × 10^3^ cells in 100 μl incomplete media/per well (media without FBS) were placed in 96-well plate and kept overnight in CO_2_-incubator. Then, the cells were treated with various concentrations of different DTX formulations (equivalent to 5–150 nM drug). After 48 h of treatment, incomplete media was discarded and 100 μl of MTT solution (1 mg/ml) was added in each well and kept in the incubator for 4 h. After incubation, MTT solution was discarded and 100 μl DMSO was added in each well to dissolve insoluble formazan dye produced by mitochondrial reductase enzyme of viable cells. Then, plates were put on shaker for 10 min and optical density (O.D.) was measured at 560 nm by plate reader (Biorad, Hercules, CA). Antiproliferative effect was evaluated by measuring the percentage of cell viability as given below:

$$\text{\% of cell viablity}=\frac{\text{O}.\text{D}.\text{at }560\text{ nm of the sample of treated cells}}{\text{O}.\text{D}.\text{at }560\text{ nm of the sample of untreated cells}}\times 100$$

#### Cellular uptake studies in C6 rat glioma cells

Cellular uptake study was performed to evaluate cellular localization of DTX-loaded fluorescent liposome in C6 rat glioma cell line (Venkatesan et al., 2011). In brief, C6 cells were seeded (3 × 10^4^) and grown on poly-L-lysine coated cover slips. Then, cells were treated with F-DTX (FITC-L-DTX) suspension in water for injection (WFI) at an equivalent DTX concentration of 7.5 nM for 0.5 and 6 h in serum free DMEM medium. After the time dependent treatment, medium was discarded and cover slips were carefully washed with PBS. Treated cells were then fixed with 4% paraformaldehyde solution for 5 min. After fixation, the cells were washed with PBS and stained with DAPI. Cover slips were dried for overnight and mounted on glass slide using DPX (dibutylphthalate polystyrene xylene). Fluorescence images (20 × magnification) were taken using a fluorescence microscope (Carl Zeiss, Oberkochen, Germany) to evaluate localization of nanoliposomes. Further, the quantification of amount of DTX uptake was done using flow cytometery analysis as follows: C6 rat glioma cells were grown in 60 mm petri dish. After 70% confluency, complete DMEM medium was removed and incomplete DMEM media was added. Then, cells were treated with F-DTX for 0.5 and 6 h. After treatment, cells were collected by trypsin treatment. Cells were then fixed in chilled ethanol and kept in −20 °C overnight. Next day, samples were centrifuged to collect the cells and resuspended in sterile PBS. Samples were then subjected to flow cytometric analysis (FACS Canto II™ cell sorter, BD Biosciences, San Jose, CA) using FACS Diva software (BD Biosciences) to measure cellular uptake of liposomes (Baishya et al., 2016).

#### In vivo plasma and brain pharmacokinetic (PK) study

*In vivo* plasma and brain pharmacokinetics of the different preparation of DTX were investigated to know the comparative distribution of DTX in the plasma and brain from L-DTX, Taxotere®, the marketed DTX formulation (Taxotere®) and free-drug suspension, consisted of 2 mg/mL of DTX in WFI (Dou et al., 2014; Dey et al., 2016).

The animals were divided into four groups. One group of animals was treated with L-DTX dispersed in WFI, second group with Taxotere® and third group with free-drug suspension in WFI at an equivalent dose of 10 mg/kg body weight of rats of DTX intravenously through tail vein (Venishetty et al., 2013; Dey et al., 2016) and the fourth group of animals was treated as control group. At a time interval of 0.25 h, 0.5 h, 1 h, 2 h, 4 h, 8 h of dosing, about 1.0 mL blood was collected into a microcentrifuge tube containing EDTA solution from the heart of each animal by terminal cardiac puncture following deep anesthesia using chloroform. The plasma was separated out by centrifugation at 5000 rpm for 6 min. The plasma was then stored at −80 °C until analysis. The brain portion was separated and stored in −80 °C until further analysis by tandem liquid chromatography-mass spectroscopy (LC-MS/MS) (Kuppens et al., 2005; Venishetty et al., 2013).

#### Sample analysis by LC-MS/MS

Working stock of DTX was prepared by serial dilution in HPLC grade methanol. Calibration control (CC) and quality control (QC) samples were prepared by spiking the working stocks in blank plasma. CC, QC and pharmacokinetic (PK) study samples were extracted by protein precipitation technique. Plasma sample (100 μl) was precipitated with 300 μl ice cold acetonitrile containing 500 ng/ml paclitaxel as internal standard (IS), vortex-mixed for 10 min, centrifuged at 4000 rpm at 15 °C for 15 min. Supernatant (100 μl) was mixed with 100 μl water and loaded into LC-MS/MS (LC: Shimadzu Model 20AC, MS: AB-SCIEX, Model: API4000, Software: Analyst 1.6) (Hou et al., 2004). Analytes were eluted using YMC Triat C18 column (2.1 × 30 mm, 5 μ) and gradient elution technique of two mobile phases (mobile phase A: 0.1% formic acid in water and mobile phase B: 0.1% formic acid in 80:20 acetonitrile/water), with injection volume: 20 μl, flow rate 0.8 ml/min and total run time 3.5 min. Brain samples were weighed and four times water was added, homogenized and analyzed by LC-MS/MS following the above-mentioned technique. Quantified DTX concentrations were plotted and different PK parameters such as maximum blood concentration (*C*_max_), area under the concentration–time curve from time of injection (*t* = 0) to a determined time point, i.e. AUC_0→_*_t_*, time taken for *C*_max_ to drop in half-life (*t*_1/2_), clearance (CL), steady state volume of distribution (*V*_ss_), mean residence time (MRT) of DTX were calculated using NCA toolbox of Phoenix-Winnonlin software (Certara, Princeton, NJ) (Kuppens et al., 2005). The intake of DTX from all the investigating formulations into the brain was assessed from the plasma to brain value (plasma/brain value) of DTX (Kemper et al., 2004; Venishetty et al., 2013).

### Statistical analysis

All the experiments were performed in triplicate in order to check the reproducibility and all the data were expressed as mean ± standard deviation. Statistical calculations were performed using one-way ANOVA followed by the Tukey *post hoc* test using Origin Pro 8 (OriginLab, Northampton, MA, USA). Differences were considered statistically significant when the probability value (*p*) was less than 0.05 at 95% confidence level.

## Results

### Drug-excipients interaction study

**Figure 1.**
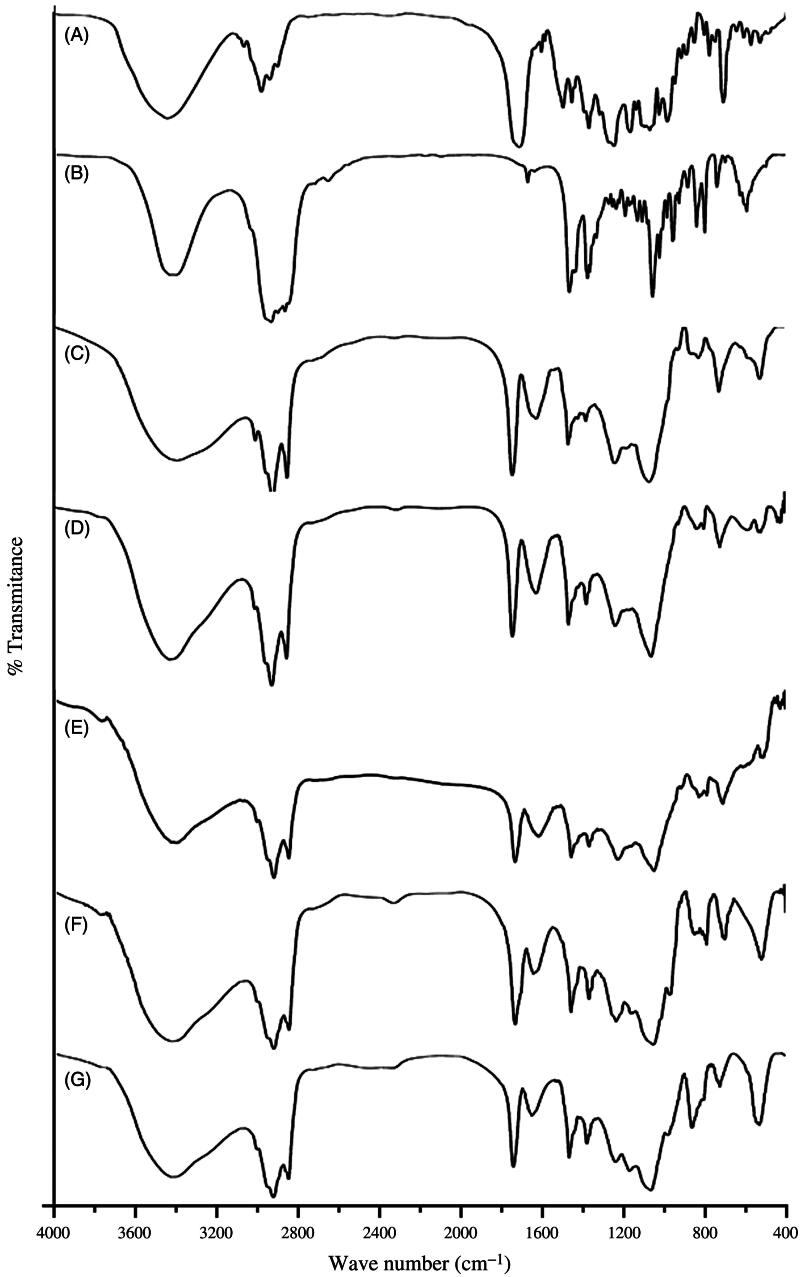
FTIR spectral data of (A) DTX, (B) CHL and (C) SPC, (D) a mixture of CHL, SPC and (E) a mixture of drug with CHL, SPC, (F) the optimized formulation with DTX (L-DTX) and (G) formulation without DTX (B-DTX).

Drug-excipients interaction (if any) was investigated using FTIR spectroscopy to assess the type of interaction among the various functional groups of the drug and the excipients of a formulation (Figure 1A–G). When the FTIR spectra of the excipients (namely, CHL, SPC) and the pure drug (DTX) were compared with their physical mixtures, it was found that the characteristic bands of all the excipients and the drug were present in their physical mixtures. The C=C asymmetric stretch of medium intensity bending vibration and C=O variable weak intensity out of plane bending vibration (at 721 cm^−1^), strong intensity C=O stretching vibration (at 1741 cm^−1^) and medium intensity bending vibration of –CH_3_ deformation (at 1379 cm^−1^) of SPC were present in the physical mixture. Similarly, the characteristics bands of CHL were observed in the spectra of physical mixture and in the spectra of cholesterol alone. For example, strong intensity stretching vibration of C–OH group (at 1057 cm^−1^) and medium intensity bending vibration of –CH_3_, –CH_2,_ –CH deformations (at 1465 cm^−1^) indicate the presence of CHL in the physical mixture of CHL, SPC and DTX and that of SPC and CHL. Further, in case of DTX, medium intensity out of plane bending vibration C=C (at 800 cm^−1^) and C–O stretching bands were observed in the spectrum of physical mixture and the spectrum of drug alone. This suggests that there is no chemical interaction seen between the drug and the excipients.

In the FTIR spectra of L-DTX and B-DTX, minor shifts of few characteristic bands of SPC from 1739 to 1743 cm^−1^ and from 1378 to 1381 cm^−1^ were observed. Again, for CHL, a shift of characteristic bands from 1465 to 1466 cm^−1^, from 1060 to 1063 cm^−1^ in L-DTX were observed. In case of DTX, the characteristic peak at wave number 800 cm^−1^ was retained by the formulation with reduction of band intensity, suggesting that there was no chemical interaction between the drug and the excipients. Further, minor shifts of some bands in case of SPC and CHL in the formulation might be due to some physical interactions. After the selection of drug and the excipients, various formulations were prepared and the best optimized formulation (NL2) was selected and reported here.

### Vesicles characterization

**Figure 2.**
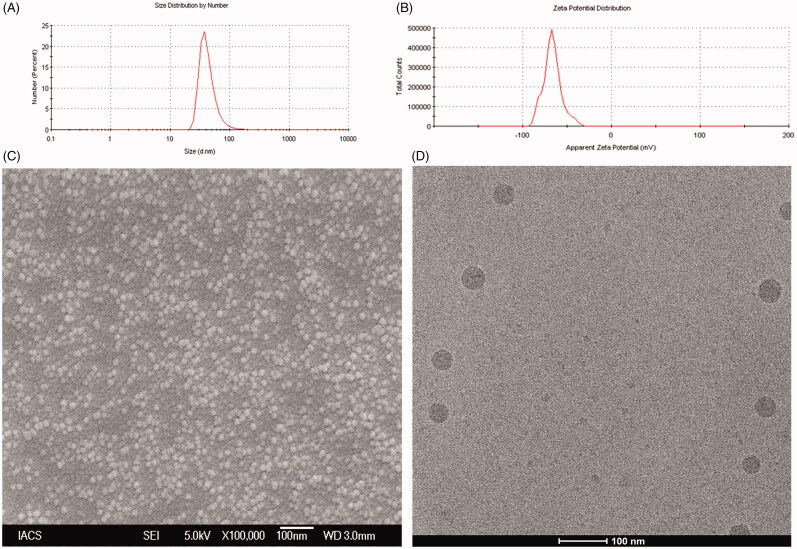
(A) Particle size distribution (B) Zeta potential value (C) Field emission scanning electron microscopic image at a magnifications of (70 000×) and (D) Cryo-TEM image of the experimental formulation (L-DTX).

The data from Malvern particle size analyzer revealed that the 100 percentage vesicles of NL2 had an average particle size 45.9 ± 12.3 nm (Figure 2A) with a PDI of 0.27 ± 0.04 and zeta potential was found to be −56.8 ± 8.7 mV (Figure 2B and Supplementary Table 1A). Further, FESEM image revealed that L-DTX had smooth surface and were homogenously distributed having size less than 50 nm (Figure 2C). From Cryo-TEM images, it was further confirmed that the vesicles were unilamellar with the intact lamellarity and were in the nanometer range (Figure 2D).

### Percentage of drug loading, drug loading efficiency percentage and yield

The percentage of drug loading of NL2 was 5.48 ± 0.5% with a drug loading efficiency percentage 87.81 ± 7.82% and product yield, 69.21 ± 4.75% (Table 1).

### *In vitro* drug release and drug release kinetic study

**Figure 3:**
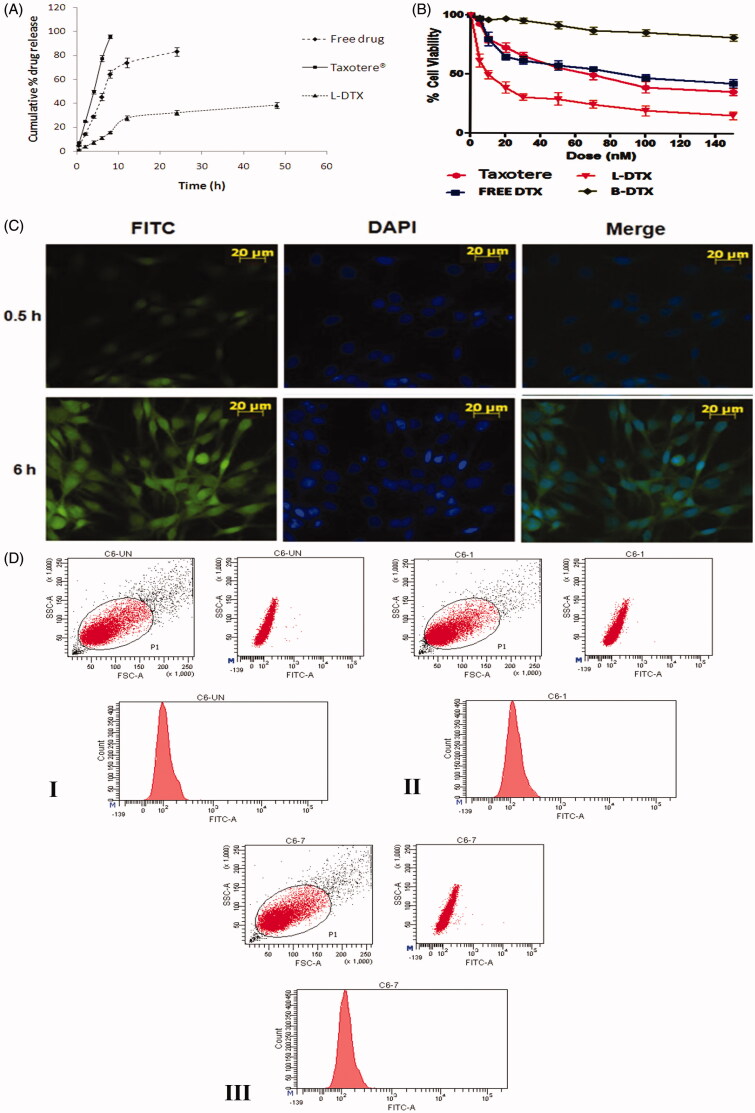
(A) *In vitro* release profile of DTX from the experimental formulation (L-DTX), marketed formulation (Taxotere®) and free-DTX solution in phosphate buffer, pH 7.4. Data show mean ± standard deviation of three different experiments in triplicate. (B) Cell viability study by MTT assay of blank formulation (B-DTX), L-DTX (formulation with DTX), marketed preparation (Taxotere®) and free-drug (DTX) in C6 glioma cells of rats. Data show mean ± standard deviation of three different experiments. (C) Cellular localization study of FITC-L-DTX (F-DTX) at different time points by fluorescence microscopy in C6 glioma cells of rats and (D) Flow cytometric measurement of C6 glioma cells of rats incubated with F-DTX at 0.5 h and 6 h of treatments, indicating about 18%, and about 23% enhancement of uptake in terms of FITC incorporated liposomes in cells at 0.5 h (Figure 3D-II) and 6 h (Figure 3D-III) of treatments, respectively, in comparison to untreated cells (Figure 3D-I).

The comparative *in vitro* release of DTX from the experimental formulation (L-DTX), Taxotere® and free-drug solution was done by dialysis method and the result was expressed as cumulative drug release percentage against time in hour (h) (Figure 3A). It shows that 38.3 ± 2.6% of drug was released at the end of 48 h, whereas the release of DTX from Taxotere® was very fast in comparison to L-DTX. More than 95% of DTX released within 8 h. The release of DTX from free-drug solution was at a lower rate than Taxotere® but quicker than L-DTX. More than 83% DTX released within 24 h. Thus the experimental formulation possessed a slower rate of release of DTX than Taxotere® and free drug solution. However, the drug released from L-DTX formulation initially at a faster rate up to 12 h and then at a slower rate in a sustained manner.

The drug release kinetic revealed that the release was occurring by the Korsmeyer–Peppas kinetic (*R*^2 ^=^ ^0.955) (Supplementary Table 2A). The release of the drug from the vesicles followed non-Fickian diffusion kinetics, as depicted from the release component value (*n* = 0.848).

**Table 2.** Pharmacokinetic parameters and plasma to brain values in different groups of rats treated with DTX-loaded liposome (L-DTX), marketed formulation of DTX (Taxotere®) and free-drug (DTX), administered intravenously at a dose of 10 mg/kg of average body weight of the rats.

Formulation	*t_1/2_ (h)	**C_max_ (ng/ml)	^#^AUC_0→_*_*t*_* (ng.h/ml)	^##^AUMC_0→_*_*t*_* (ng.h2/ml)	^$^MRT (h)	^+^CL (L/h)	^++^*V*_ss_ (L)	Mean plasma/brain value at different time interval in hour	
L-DTX	1.97 ± 0.02^¶^^,^^€^	1508.4 ± 33.9	2061.04 ± 88.19^¶^	3630.19 ± 255.81^¶^^,^^€^	1.76 ± 0.13^¶^^,^^€^	4.85 ± 0.26^¶^	8.55 ± 0.49^¶^^,^^€^	0.5	0.016 ± 0.001
								1.0	0.058 ± 0.005^¶^
								2.0	0.059 ± 0.008
								4.0	0.072 ± 0.017
Taxotere®	0.89 ± 0.05^€^	1725.2 ± 179.1^¥^	2192.57 ± 168.80^¥^	2726.41 ± 431.27^€^^,^^¥^	1.24 ± 0.08^€^	4.56 ± 0.22^¥^	5.67 ± 0.29^¥^^,^^€^	0.5	0.019 ± 0.003
								1.0	0.063 ± 0.001^¥^
								2.0	0.049 ± 0.005
								4.0	0.035 ± 0.0038
Free drug	0.81 ± 0.04^¶^	1308.1 ± 54.7^¥^	1499.25 ± 68.73^¶^^,^^¥^	1587.77 ± 206.70^¶^^,^^¥^	1.06 ± 0.08^¶ ^	6.67 ± 0.30^¶^^,^^¥^	7.06 ± 0.17^¶^^,^^¥^	0.5	×××
								1.0	0.0143 ± 0.002^¶^^,^^¥^
								2.0	×××
								4.0	×××

Values represent mean ± standard deviation (*n* = 3); Statistical calculations were performed using one-way ANOVA followed by the Tukey *post hoc* test using Origin Pro 8 (OriginLab, Northampton, MA). Differences were considered statistically significant when the probability value (*p*) is less than 0.05 at 95% confidence level.

* *t*_1/2_, half-life.

** *C*_max_, maximum blood concentration.

^#^ AUC_0→_*_t_*, area under the concentration-time curve from time of injection (t = 0) to a determined time point.

^##^ AUMC, area under the first moment curve.

^$^ MRT, mean residence time.

^+^ CL, clearance.

^++^ *V*_ss,_ steady state volume of distribution.

^¶^ Denotes comparison made between L-DTX and free drug.

^€^ Denotes comparison made between L-DTX and Taxotere®.

^¥^ Denotes comparison made between Taxotere® and free drug.

### *In vitro* cell viability assay

*In vitro* cell viability or anti-proliferative effect of free DTX, L-DTX, B-DTX and Taxotere®, was evaluated in C6 glioma cells of rats using MTT assay method. The plot of % cell viability against dose (nM) shows that as the concentration of formulation of DTX (L-DTX) increased, the death rate of cells increased. L-DTX-mediated cellular death was found to be more compared to the cells treated with free-drug solution and Taxotere®, respectively. The half maximal inhibitory concentration (IC_50_) value of L-DTX significantly decreased in comparison to others. IC_50_ values of DTX from L-DTX was found at 9.5 ± 0.8 nM which is significantly very less in comparison to free-DTX (IC_50_ value, 70.8 ± 0.1 nM) and Taxotere® (IC_50_ value, 86.5 ± 0.3 nM). The data also reveal that there was no effect of the excipients used in the formulation on the cytotoxicity of DTX as the cell death from blank formulation (B-DTX) was insignificant (Figure 3B).

### Cellular uptake studies in C6 rat glioma cells

To ascertain whether the prepared NLs can permeate into the cell or not, we have performed *in vitro* cellular uptake study in C6 glioma cells by fluorescent microscopy using F-DTX formulation. The images reveal that the formulation was internalized into the cells through the cell membrane and distributed at the cytoplasm. As the time of dosing increased, the amount of uptake of NLs also increased (Figure 3C). This was further substantiated by the flow cyclometry study (Figure 3D), data shows about 18%, and about 23% enhancement of uptake in terms of FITC incorporated liposomes in cells at 0.5 h (Figure 3D-II) and 6 h (Figure 3D-III) of treatments, respectively, in comparison to untreated cells (Figure 3D-I).

### *In vivo* plasma and brain pharmacokinetic (PK) study

The pharmacokinetic data of DTX from the experimental formulations in plasma and brain were investigated in Sprague-Dawley rats. A dose of 10 mg/kg body weight of rats of DTX was administered by intravenous route and different PK parameters of DTX were calculated (Table 2).

**Figure 4:**
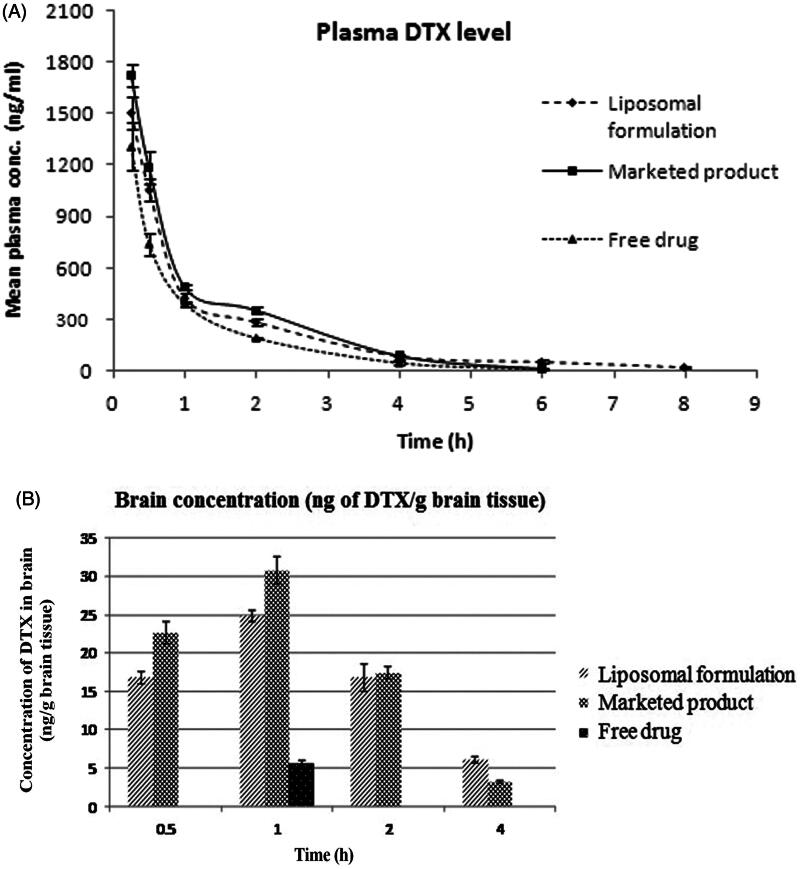
(A) Plasma level of DTX in rats after intravenous administration of L-DTX, free drug and Taxotere®. (B) Concentration of DTX in brain after intravenous administration of nanoliposomal formulation containing DTX (L-DTX), marketed formulation (Taxotere®) and free drug to rats at a dose of 10 mg/kg of DTX. Note: Data show mean ± standard deviation (*n* = 3).

The mean plasma concentrations of DTX from L-DTX at the different time points were comparatively higher than those of the free DTX-treated rats up to 6 h (Figure 4A). However, the plasma concentration of DTX could not be detected below the lower limit of quantification (2 ng/mL) after 6 h for the free-drug, but for L-DTX, the drug was detected up to 8 h. The plasma concentration of DTX from L-DTX was 5.44-fold higher than that of free drug at the end of 8 h, indicating more circulation of DTX in the blood of the experimental rats.

When we compared Taxotere® with L-DTX, concentrations of DTX in plasma at the different time points of post-dosing did not significantly vary. The plasma t_1/2_ value of DTX from L-DTX was higher than those of the free-drug and Taxotere®-treated rats by 2.43 folds and 2.21 folds, respectively. MRT value of DTX was considerably higher for L-DTX than free-drug and Taxotere® by about 66% and 42%, respectively. Further, there was a decrease of clearance and increase of AUC of DTX by 27.3% and 37.5%, respectively, in L-DTX-treated rats in comparison to free-drug-treated group of animals.

DTX level in brain was 4.4-fold higher in L-DTX-treated rats than the free-drug-treated rats at 1 h (Figure 4B). After 1 h the drug concentration in brain could not be quantified for free-drug-treated animals although DTX concentration for L-DTX-treated animals was measured up to 4 h. The drug level in brain from L-DTX was little lower than that from Taxotere® up to 2 h, but DTX concentration became predominantly more in L-DTX-treated animals at 4 h than Taxotere®-treated rats.

We have also calculated the plasma/brain value of DTX from the different groups of animals at the different time points up to 4 h of study. There was 4.07-fold increase of plasma/brain value of DTX for L-DTX with respect to the free-drug-treated animals at 1 h. The values of plasma/brain of DTX for L-DTX and Taxotere® were 0.0159 vs. 0.0190 at 0.5 h, 0.0583 vs. 0.0628 at 1 h, 0.0589 vs. 0.0487 at 2 h, respectively. At 4 h, the value from L-DTX was predominantly higher than that of Taxotere® (Table 2).

## Discussion

NLs were prepared by lipid layer hydration technique, and the drug and the excipients were taken at the ratio (by weight) of DTX, SPC, CHL varied from 1:5:5 (NL1) to 1:10:5 (NL2) to 1:15:5 (NL3) (w/w/w) (Table 1) to get optimized formulation in terms of better product yield, percentage of drug loading and drug loading efficiency percentage. The percentage of drug loading and percentage loading efficiency study revealed that 5.48 ± 0.5 μg of drug per mg of the formulation with a loading efficiency of 87.81 ± 7.82% was achieved in case of NL2 formulation. So, a ratio of 1:10:5 of DTX:SPC:CHL was considered as an optimized formulation in this work. Hence, it has been considered for further physicochemical characterization and different *in vitro* and *in vivo* pharmacokinetic studies.

DTX is an approved drug to tumor of brain in combination therapy, but its entry to the brain is prevented by BBB due to its different physiochemical and pharmacological factors (Chen et al., 2004; van Rooy et al., 2011).We have tried to deliver DTX by incorporating it into the liposomes, based on the hypothesis that the nanosize of the vesicle may help to deliver DTX and the use of nanoliposomes may overcome the solubility problem of DTX and reported toxicity of Taxotere®, the marketed formulation (Yang et al., 2007; Yousefi et al., 2009; Costantino & Boraschi, 2012). In this work we have formulated nanoliposomes of DTX using SPC as lipid component, and CHL as a stabilizer of lipid membranes.

Drug-excipients interaction was studied by FTIR spectroscopy. FTIR spectra showed that the characteristic peaks of drug, SPC and CHL were present in the physical mixture of the excipients and drug; and in the formulation. The characteristic peak of DTX existed in the formulation suggests that drug was encapsulated in the bilayer.

The FESEM images of the optimized formulation show that the obtained vesicles were within 50 nm in size and had smooth surface, and they were homogenously distributed. Further, their characterization by Cryo-TEM reveals that the obtained NLs were of unilamellar spherical vesicle with intact lamellarity.

The particle size analysis data by DLS method show that the NLs were in the nanometer range with a very narrow size distribution as indicated by the PDI value. Further, PDI value of a colloidal system gives an indication of particle size distribution and also the physical stability of the system. For drug delivery aspects by intravenous injection or intravenous infusion, it is preferred that the particle should be of same size in the formulation. The PDI value of 0.1–0.25 indicates that the liposomal vesicles are uniformly distributed having appreciable physical stability and PDI value more than 0.5 indicates poor uniformity of the vesicles in the formulation (Dey et al., 2016). In this study, we obtained nanoliposomes having PDI value 0.27 ± 0.04, indicating that the prepared liposomes were mostly distributed homogenously.

The physical stability of the vesicles can also be predicted from the value of zeta potential. A zeta potential value above +30 mV and/or below −30 mV indicates that the vesicles of liposomes are good as per stability of the colloidal system is concerned (MacLachlan , 2007; Dey et al., 2016). In this work we got vesicles having zeta potential 56.8 ± 8.7 mV, suggesting that L-DTX vesicles had good colloidal stability. The presence of terminal carboxyl group in the lipids could be liable for this negative charge in the NLs.

*In vitro* drug release profile of the optimized formulation shows that the release of DTX from the experimental formulation (L-DTX) was found to occur faster initially (up to 12 h) and then at a slower rate in a sustained manner in comparison to free-drug and marketed product.

The pattern of drug release from the formulation was best fitted with the Korsmeyer–Peppas kinetics, which clearly demonstrates the involvement of anomalous diffusion which is controlled by more than one parameter. In case of the Korsmeyer–Peppas model, the fraction of drug release with time is considered and represented as *M_t_*/*M*_∞_ = *Kt*^n^, where *M_t_*/*M*_∞_ is a fraction of drug released at time *t* and *n* is the drug release exponent. The drug release mechanism is governed by the value of “*n*”. When *n* ≤ 0.45, the drug release is considered to follow the Fickian diffusion mechanism and it is non-Fickian when *n* = 0.45–0.89 (Pattnaik et al., 2012). When *n* = 0.89, drug release follows Case II (relaxational) transport, and if *n* > 0.89, it undergoes super case II transport mechanism of drug release (Pattnaik et al., 2012). The value of “*n*” was 0.848 in our study. Hence, the drug release occurred by non-Fickian diffusion or anomalous diffusion mechanism for L-DTX.

The MTT assay data for antitumor activity assessment in C6 rat glioma cells reveal that at each dosing interval the death rate was always higher for L-DTX as compared to Taxotere® and free-drug, suggesting that better antitumor efficacy of the experimental nanoliposomal vesicles of DTX (L-DTX) was seen at a comparatively low dose and the IC_50_ value of L-DTX was significantly decreased (*p* < 0.05) as compared to Taxotere® and free drug-treated C6 cells. For B-DTX formulation (without drug), the decrease in cell viability of the cultured cell population was not notably significant, suggesting that the excipients of the formulation had no predominant impact on the death rate and these excipients are safe for glioma treatment.

For cellular uptake of NLs, C6 glioma cell line was used. FITC-NLs were used to visualize localization of the vesicles in the cells by its green fluorescence. In order to discriminate the localization of the vesicles into the nucleus, cells were stained with DAPI, as a nucleus staining color substance (blue fluorescence). The fluorescence microscopy images and the flow cyclometry quantification of the internalized FITC incorporated liposomes disclosed that our nanoliposomal formulation predominantly concentrated in a time-dependent manner in the cytoplasmic part of the cells.

Pharmacokinetic profiles of DTX in plasma and brain were evaluated after its intravenous dosing in rats at a dose of 10 mg/kg. Pharmacokinetic studies reveal that the plasma concentrations of DTX were higher for the experimental formulation at each investigational time point in comparison to free-drug. There was a rapid decrease of the plasma level up to first 2 h, which might be due to the rapid distribution of drug in the other tissues. Our formulation sustained the release of the drug at least up to 8 h (period of study) while the marketed product and the free-drug treatments showed detectable blood level of the drug up to 6 h only. There was a significant difference (*p* < 0.05) in AUC values among the various groups of animals treated with free-drug, Taxotere® and the experimental formulation. The AUC values of L-DTX and Taxotere®-treated rats were significantly higher (*p* < 0.05) than that of the free-drug treated rats, whereas there was non-significant difference (*p* > 0.05) of AUC value between L-DTX and Taxotere®.

The plasma/brain values at 1 h among the free-drug, Taxotere® and L-DTX were significantly different (*p* < 0.05), but upon the Tukey *post hoc* test, there was no significant difference in value between our formulation and Taxotere® (*p* > 0.05). Amount of drug was not detectable at 0.5 h in brain tissues from the animals treated with free-drug. However, at 1 h only, DTX was detected in the same groups of animals. The DTX concentration in brain was observed from L-DTX and Taxotere® at all the time points studied.

In L-DTX-treated rats, the plasma/brain values were more than those of the marketed formulation at 2 h and 4 h of the study. Thus the experimental formulation crossed through the BBB better into the brain compartment than the free-drug. L-DTX was found to maintain 100% more drug concentration in brain at 4 h as compared to the marketed formulation. The size ranges of nanocarriers should be from 40 nm to 200 nm, for a successful brain delivery of a drug (Jain , 2012; Masserini , 2013; Mukherjee et al., 2015; Sonali et al., 2016a; Shilo et al., 2015). The vesicle size in this work was below 50 nm, hence the experimental formulation might improve the drug delivery by virtue of its nanosize (Sonali et al., 2016a; Shilo et al., 2015; Jain , 2012; Masserini , 2013; Mukherjee et al., 2015) and highly lipidic in nature. The decrease of brain concentration of the drug with time was held faster for Taxotere® than L-DTX. The MRT value of DTX from L-DTX was significantly elevated (*p* < 0.05) than free drug and Taxotere®-treated groups of rats (Table 2). Thus, the MRT values indicate that L-DTX was available in plasma more than the marketed formulation, suggesting longer sustaining effect of the drug from L-DTX.

A comparable drug level in brain from the experimental formulation with respect to the tween 80 containing commercial formulation suggests that the use of nanoliposome may avoid tween 80-related side-effects.

## Conclusion

DTX-incorporated nanoliposomes may help us to avoid the use of tween 80, and thus escape tween 80 containing formulation related side-effects. Due to the sustained release of the drug from the vesicles, nanoliposomes of DTX may be used for depot preparation. Predominant uptake of L-DTX in the rat glioma cells showed the successful entry of the drug in the cells. The nanosize of the prepared vesicles was found to cross the BBB successfully *in vivo*. The experimentally developed nanoliposomes are thus found to be an emerging way to deliver the drug in the brain and this could be a successful strategy to treat brain cancer using DTX.

## Supplementary Material

supplementary_tables.doc

## Notes

**Supplementary material available online**
