# Supplementary material for: Successful delivery of docetaxel to rat brain using experimentally developed nanoliposome: a treatment strategy for brain tumor
Source: Drug Deliv. 2017 Feb 6;24(1):346–57. doi: 10.1080/10717544.2016.1253798 (PMC8240984; doi:10.1080/10717544.2016.1253798)
Supplement: supplementary_tables.doc [file IDRD_A_1253798_SM3817.doc]

**Supplementary Tables:**

Table 1: Particle size, PDI value and zeta potential of L-DTX

| Formulation code | Particle size* | PDI* | Zeta potential* |
| --- | --- | --- | --- |
| NL2 (L-DTX) | 45.9±12.3 nm | 0.27±0.04 | -56.8±8.7 mV |

*Mean±standard deviation (n=3)

Table 2: *In vitro* drug release kinetic equations with R^2^ values and release exponent (n) of L-DTX

| *In vitro* release kinetics | L-DTX |
| --- | --- |
| Zero order kinetic | y = 0.851x + 6.452 R² = 0.751 |
| First order kinetic | y = -0.004x + 1.972 R² = 0.799 |
| Korsmeyer-Peppas kinetic | y = 0.848x + 0.382 R² = 0.955, n=0.848 |
| Higuchi kinetic | y = 6.710x - 3.058 R² = 0.912 |
| Hixon-Crowell kinetic | y = -0.015x + 4.542 R² = 0.783 |

R^2^=Correlation coefficient,n = Release exponent
